# Importance of Demographic and Clinical Features in Evaluating the Severity of COVID-19 in Hospitalized Patients: A Serbian Retrospective Study in the First Pandemic Year

**DOI:** 10.3390/jcm12144638

**Published:** 2023-07-12

**Authors:** Jelena Aritonovic Pribakovic, Milica Peric, Aleksandra Milenkovic, Aleksandra Janicevic, Snezana Hadzistevic, Aleksandra Ilic, Mirjana Stojanovic-Tasic, Kristina Bulatovic, Dragisa Rasic, Jadranka Mitic

**Affiliations:** 1 Faculty of Medicine in Pristina, University of Pristina Temporarily Settled in Kosovska Mitrovica, 38220 Kosovska Mitrovica, Serbia; milicaperic110@gmail.com (M.P.); aleksandra.petrovic@med.pr.ac.rs (A.M.); s.hadzistevic@med.pr.ac.rs (S.H.); aleksandra.ilic@med.pr.ac.rs (A.I.); mstojanovictasic@gmail.com (M.S.-T.); kristinajakovljevic@gmail.com (K.B.); rasic_dragisa@yahoo.com (D.R.); jadrankajacamitic@gmail.com (J.M.); 2 Clinical Hospital Center Pristina, 38205 Gracanica, Serbia; aleks121292@gmail.com; 3 Clinical Hospital Center Kosovska Mitrovica, 38220 Kosovska Mitrovica, Serbia

**Keywords:** aged, comorbidity, COVID-19, disease severity, clinical features

## Abstract

The aim of this study is to determine the demographic and initial clinical characteristics of patients with COVID-19 and their importance in evaluating the severity of the disease. A retrospective study included patients suffering from COVID-19 who were hospitalized at The Department of Infectious Disease of the Clinical Hospital Center Pristina—Gracanica from the beginning of the pandemic until the end of 2020. We compared the symptoms of the disease, radiographic findings of pneumonia, laboratory parameters, duration of symptoms before admission, the difference in the need for certain therapies, and the presence of comorbidities between non-severe and severe groups of patients. Patients with a severe disease were statistically significantly older. Hypertension was significantly associated with severe clinical conditions. Radiographic findings of bilateral pneumonia on admission were much more frequent among the severe group, and these patients’ need for oxygen support was significantly higher. Lower neutrophil and higher lymphocyte counts were statistically significant in the non-severe group. Biochemical parameters at admission also showed statistical significance between the examined groups. Based on our research, we can conclude that a complete overview of the patient, including demographic and laboratory parameters as perhaps the most significant attributes, can help doctors in the timely clinical assessment of patients and, thus, in the timely application of adequate therapeutic protocols in the treatment of COVID-19.

## 1. Introduction

COVID-19 (Coronavirus disease) is an acute infectious disease caused by a new strain of coronavirus named the severe acute respiratory syndrome coronavirus-2 (SARS-CoV-2). Globally, by May 2023, there have been 766,440,796 confirmed cases of COVID-19, including 6,932,591 deaths, reported to the WHO [1]. The clinical features vary from asymptomatic and moderate to severe forms of acute respiratory distress syndrome (ARDS). Symptoms appear 2–14 days after exposure to the virus. The most common clinical features are a dry cough, elevated body temperature, fatigue, difficulty breathing, headache, muscle pain, and pneumonia. In patients with COVID-19, the severity of hypoxemia is independently associated with in-hospital mortality and may be an important predictor of the risk that the patient needs to be admitted to an intensive care unit [2], even though he does not show signs of respiratory distress or verbalize the sense of dyspnea [3]. Lack of sense of smell and taste, nausea, vomiting, diarrhea, hemoptysis, rhinorrhea, weight loss, kidney, and liver damage occur less often [4,5,6]. 

In laboratory analyses, the finding of lymphopenia with an increased neutrophil count, thrombocytopenia, and anemia is distinctive. There are also elevated values of lactate dehydrogenase (LDH), aspartate aminotransferase (AST), alanine aminotransferase (ALT), creatine kinase (CK), D-dimer, C reactive protein (CRP), and decreased albumin values [5,7]. 

Many risk factors have been identified in the progression of COVID-19 into a severe and critical stage, including old age, male gender, and underlying comorbidities such as hypertension, diabetes, obesity, lung diseases, heart, liver, and kidney disease, immunodeficiency conditions, and malignancies. In addition, socioeconomic status, diet, lifestyle, geographical differences, ethnicity, exposed viral load, day of initiation of treatment, and quality of health care have all been reported to influence individual outcomes. Furthermore, higher CRP and radiography findings at admission are associated with COVID-19 pneumonia, intensive care unit admission, and death [8,9,10,11,12]. 

The aim of this study is to determine the demographic and initial clinical characteristics of patients with COVID-19, the need for oxygen and corticosteroid therapy on admission, and the importance of these parameters in evaluating the severity of the disease.

## 2. Materials and Methods

### 2.1. Ethical Approval

The research was approved by the Ethics Committee for Scientific Research of the Clinical Hospital Center Pristina—Gracanica of the Republic of Serbia.

### 2.2. Data Collection

This retrospective study included 330 patients with confirmed SARS-CoV-2 infections who were hospitalized at the Department of Infectious Disease of the Clinical Hospital Center Pristina—Gracanica from the beginning of the pandemic until the end of 2020. Persons under the age of 18, pregnant women, PCR-negative patients, and patients with incomplete results were excluded from the study.

The demographic and health data of the patients, PCR and blood tests, and plain radiographs of the lungs on admission were obtained from the database of the hospital’s information system. 

The COVID-19 diagnosis was confirmed by a positive PCR test (Novel Coronavirus (2019-nCoV) Nucleic Acid Diagnostic Kit (PCR-Fluorescence Probing), Sansure Biotech, People’s Republic of China), which detected nucleic acid from a nasopharyngeal swab sample.

Blood tests obtained by standard laboratory methods included: complete blood count; C reactive protein (CRP); aspartate aminotransferase (AST); alanine aminotransferase (ALT); alkaline phosphatase (ALP); gamma glutamyl transferase (GGT); amylase; lactate dehydrogenase (LDH); proteins; albumin; glucose; and creatine kinase (CK). 

All patients suffering from COVID-19 were treated according to the therapeutic protocol of the Republic of Serbia for the treatment of patients with COVID-19.

Based on the development of the disease during hospitalization, the patients were categorized into two groups, including the non-severe (mild/moderate disease) and severe (severe/critical disease) groups, in accordance with the criteria of the World Health Organization [13]. Non-severe cases had either mild clinical symptoms without signs of pneumonia on imaging (mild type) or fever and respiratory symptoms with signs of pneumonia on imaging (moderate type). Cases in the severe group met at least one of the following criteria: respiratory rate ≥ 30/min; oxygen saturation ≤ 93%; the ratio of arterial partial oxygen pressure to inspiratory oxygen fraction (PaO_2_/FiO_2_) ≤ 300 mmHg; respiratory failure requiring mechanical ventilation; and shock or other organ failure requiring intensive care support. We then compared the symptoms of the disease, radiographic findings of the lungs, laboratory parameters, duration of symptoms before admission, differences in the need for certain therapies, and the presence of comorbidities between these two groups.

### 2.3. Statistical Analysis

Data are presented with descriptive parameters: median (Min-Max) value and absolute and relative frequencies. Numerical data were tested with the Shapiro–Wilk test, and because they did not have a normal distribution, the difference in mean values was tested with the non-parametric method of the Mann–Whitney U rank sum test. The chi-square test was used to test the hypothesis about the difference in frequencies. All variables that were statistically significant at the level of univariate analysis were included in the model of multivariate regression analysis, and binomial logistic regression was applied with severity of disease as the dependent variable.

The criterion for statistical significance was *p* < 0.05. The software program SPSS Statistics 22 (SPSS Inc., Chicago, IL, USA) was used for statistical analyses. 

## 3. Results

The study included 330 patients, ages 18 to 92. The average age was 57.0 ± 16.4. Of the total number of patients, 192 (58.2%) were male and 138 (41.8%) were female. The median age of the men was 62 (range 23.0–86.0), and the median age of the women was 54.5 (range 18.0–92.0). The male gender is statistically significantly older (*p* = 0.015). A total of 231 (70.0%) patients had a non-severe disease and 99 (30.0%) had a severe disease. Patients with a severe disease were statistically significantly older (*p* < 0.001). The average age of patients in the non-severe group was 55.0 (18–92) years, while the average age of patients in the severe group was 65.0 (23–92) years. 

**Figure 1:**
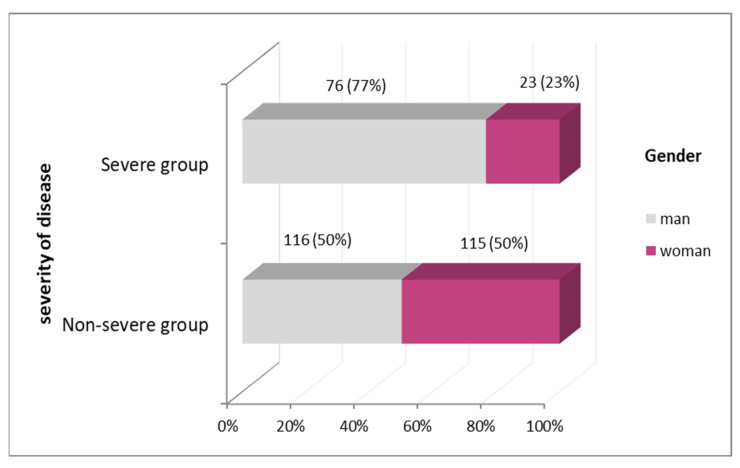
Gender distribution according to the severity of the disease.

The frequency of gender is statistically significant for the treatment outcome (chi-square = 20.079, *p* < 0.001) since the male gender is statistically significantly more frequent in the group of patients whose disease was severe (Figure 1).

The symptoms that the patients had on admission (cough, shortness of breath, fatigue, headache, anosmia, and gastrointestinal complaints) did not show a statistically significant difference between the groups. The most common symptom was cough, which was present in 76.5% of patients, and the least common was anosmia, which was present in 10.2% of patients. Hypertension was significantly more frequent in patients with a severe disease (*p* = 0.022), while diabetes mellitus and chronic obstructive pulmonary disease did not show any statistical significance between groups. Pneumonia, as well as bilateral pneumonia compared to unilateral pneumonia on admission, was statistically significantly more present in patients with a severe disease (*p* < 0.001) (Table 1). 

The duration of symptoms before admission, as well as the presence of elevated body temperature, did not show a statistically significant difference between the examined groups (*p* = 0.926; *p* = 0.352) (Table 2). 

Regarding the laboratory parameters, in the blood count, there was a statistically significant difference in the neutrophil and lymphocyte counts. Lower neutrophil and higher lymphocyte counts were statistically significant in the non-severe group (*p* < 0.001). Biochemical parameters at admission, CRP, AST, ALP, LDH, glucose, proteins, albumin, and CK showed statistical significance between the examined groups (*p* < 0.001). The oxygen saturation on admission was statistically significantly lower in patients who developed a severe disease (Table 2) (*p* < 0.001), and therefore the need for oxygen support already on admission was significantly more frequent in that group (*p* < 0.001). The use of corticosteroids was more frequent in patients with a severe disease (65.7% vs. 54.1%) (Table 3).

All variables that were significant at the level of univariate analysis were included in the multivariate logistic regression model. The model contains 15 predictors and is statistically significant (chi-square = 57.819, *p* < 0.001). CK (B = 0.002, *p* = 0.032) and oxygen support (B = 2.178, *p* < 0.001) stood out as significant predictors of the severity of disease. Oxygen support is a strong predictor of severe forms of disease. It shows that patients in need of oxygen therapy on admission to the hospital had a nearly nine-fold higher risk of developing a more severe form of the disease. CK stood out as a significant predictor, but it does not greatly increase the chance of a more severe form of the disease (OR = 1.002) (Table 4).

## 4. Discussion

Like the majority of research [8,14], our results showed that severe disease was significantly more frequent in elderly male patients. However, on the contrary, Zhang X et al. have said that there is no difference in gender or age between patients with mild, moderate, or severe disease [15]. Possible explanations for our results include a higher frequency of comorbidities associated with aging. Moreover, the significant influence of gender on the severity of the disease in our study could be explained by different male and female presentations, different structures of patients (in terms of representation of age, comorbidities, and habits according to gender), as well as the limited number of patients included in the study. 

Of the symptoms of patients on admission, the most common was cough, with 76.5%, followed by fatigue, with 66.0%, while other symptoms (headache, shortness of breath, and gastrointestinal symptoms) were present in less than 50% of patients who required hospitalization. We can explain this by the fact that COVID-19 emerged mainly as a respiratory disease. Similar to our results, the results of Egyptian researchers have shown that the most common symptoms were fever and cough [16,17], while the other symptoms were present in less than 50% of patients [18]. 

The duration of the symptoms before admission to the hospital did not have an influence on the severity of the disease, which is contrary to the results of Loss SH et al. [18] and Stojanovic M et al. [19]. Our results can be explained by the fact that some of our patients were initially treated at home. In addition, some patients ignored the first symptoms of the disease.

Comorbidities are considered risk factors for severe cases of COVID-19 [4,8]. Our results show that hypertension is more frequent in patients with severe disease, which is in agreement with the results of Allwood BW et al. [20]. In contrast to Los et al. [18], our results show that diabetes and chronic obstructive pulmonary disease did not have a statistically significant difference between the two groups. Although in our study diabetes did not prove to be a statistically significant comorbidity, we note that patients who had significantly higher glucose values at admission had more severe clinical conditions. It is possible that among those patients were also patients with previously undetected diabetes. This could explain the inconsistency of our results with those of the previously mentioned authors.

Oxygen saturation on admission was the best predictor of mortality in examined patients aged 18 to 50 years [21]. Furthermore, our results showed that patients with significantly lower oxygen saturation and the consequent need for oxygen support already on admission had a severe disease. 

Among the laboratory abnormalities, the most common is the occurrence of lymphopenia with an increased number of neutrophils, thrombocytopenia, anemia, and elevated values of LDH, AST, ALT, CK, D-dimer, CRP, and decreased albumin values [5,7,22]. Laboratory markers associated with increased risk for severe disease include lymphocytopenia, neutrophilia, and elevated serum ALT, AST, LDH, CRP, and ferritin [23,24]. Furthermore, our results agree with the majority of authors [5,7,21,25] in that lymphopenia and elevated values of AST and LDH were statistically significantly more frequent in patients with severe forms of the disease. We can assume that a reduced number of lymphocytes is a consequence of viral attachment, damage to the immune system, or exudation of circulating lymphocytes into the inflammatory lung tissue. The increased values of LDH and AST can be attributed to greater cytotoxic damage in clinically severe cases. Moreover, in our patients, there is a statistically significant difference in ALP values between the groups, which was significantly lower in patients with severe disease. While the results of Turkish researchers show that, except for saturation, both high CRP and low albumin are good predictors of mortality [21], our results also show that high CRP, as well as low albumin and hypoproteinemia on admission, show significant differences between patients who had mild/moderate and severe disease. Our results are in agreement with other authors whose research shows that high CRP and low albumin values are important for the development of a severe form of the disease [26,27], indicating the development of cytokine storms in patients with COVID-19. Furthermore, our patients with severe disease already had significant elevated CK on admission. Parameters reported at the time of admission can indicate to the doctor the possible development of a severe disease in the patient and thus an increased level of caution in the treatment approach. 

The use of corticosteroids and the need for oxygen support already on admission were more frequent in the group of patients with severe disease. 

In our research, the strongest predictor of the development of a severe form of the disease is the need for oxygen at admission. Patients who required oxygen therapy on admission to the hospital had an almost nine-fold greater risk of developing a more severe form of the disease. Creatin kinase also stood out as a significant predictor, but it does not greatly increase the chance of a more severe form of the disease, which is similar to the results of Friedman et al. [28]. Secondary systemic myositis as a direct consequence of coronavirus infection may be a reasonable explanation for this increase in CK levels [29,30].

## 5. Conclusions

Based on our research, we can conclude that a complete overview of the patient, including demographic data, comorbidities, pneumonia, laboratory parameters, and, most significantly, the need for oxygen support at admission, can help doctors in the timely clinical assessment of patients and hence in the timely application of adequate therapeutic protocols in the treatment of COVID-19.

## 6. Study Limitation

Our study has a few limitations. First, this was a retrospective study. Second, our study included more hospitalized patients with mild and moderate forms of COVID-19 (non-severe group) than those with severe and critical cases (severe group). Third, the current analysis was restricted to a specific geographical region, which could reduce the generalizability of our findings.

## Figures and Tables

**Table 1:** Clinical characteristics on admission.

	Characteristics of Patients		Non-Severe Group	Severe Group	Significance of *p* Value
	Number of Patients *n (%)*		231 (70.0%)	99 (30.0%)	
Symptoms	Cough *n* (%)		170 (73.6%)	82 (82.8%)	0.070
	Fatigue *n* (%)		153 (66.2%)	64 (64.4%)	0.781
	Headache *n* (%)		62 (26.8%)	23 (23.2%)	0.492
	Gastrointestinal symptoms *n* (%)		42 (18.2%)	19 (19.2%)	0.829
	Anosmia *n* (%)		27 (11.7%)	7 (7.1%)	0.206
Comorbidities	Hypertension *n* (%)		50 (21.6%)	33 (33.7%)	0.022 *
	Diabetes mellitus *n* (%)		27 (11.7%)	14 (14.3%)	0.514
	Chronical obstructive pulmonary disease *n* (%)		5 (2.2%)	3 (3.1%)	0.629
Radiographic findings	Radiographic finding of pneumonia *n* (%)		182 (79.1%)	91 (94.8%)	0.001 *
	Localization of pneumonia *n* (%)	Unilateral	52 (28.6%)	5 (5.5%)	0.001 *
		Bilateral	130 (71.4%)	86 (94.5%)	

* Statistically significant value.

**Table 2:** Clinical and laboratory parameters on admission.

Laboratory Parameters	Non-Severe Group	Severe Group	Significance of *p* Value
	Median (Min–Max)	Median (Min–Max)	
Body temperature (°C)	37 (35–40)	38 (36–40)	0.373
Duration of symptoms before admission(days)	5 (1–30)	5 (1–30)	0.841
Leukocytes (×10^9^/L)	6.30 (2–26)	6.20 (2–48)	0.967
Neutrophils (%)	0.70 (0.23–0.93)	0.79 (0.10–0.95)	0.001 *
Lymphocytes (%)	0.26 (0.04–0.61)	0.18 (0.05–0.84)	0.001 *
Erythrocytes (×10^12^/L)	4.5 (2.92–5.98)	4.49 (2.93–5.70)	0.591
Hemoglobin (g/L)	139 (89–185)	139 (89–176)	0.907
Hematocrit (L/L)	0.44 (0.29–0.57)	0.44 (0.30–0.55)	0.839
Platelets (×10^9^/L)	228 (70–989)	205 (73–442)	0.123
CRP (IU/mL)	24 (3–145)	34 (6–150)	0.008 *
AST (U/L)	42 (15–241)	51.5 (21–220)	0.037 *
ALT (U/L)	42 (10–260)	44 (12–258)	0.669
ALP (U/L)	108 (59–303)	99 (55–486)	0.027 *
GGT (U/L)	31.5 (10–256)	32 (12–258)	0.288
Amylase (U/L)	56 (13–262)	49 (16–150)	0.286
LDH (U/L)	415 (194–1399)	563.50 (247–2081)	0.001 *
CK (U/L)	92 (16–3285)	145 (22–1956)	0.001 *
Total proteins (g/L)	72 (51–98)	69 (48–86)	0.001 *
Albumin (g/L)	42 (25–82)	36.50 (20–80)	0.001 *
Glucose (mmol/L)	6.64 (4–10)	7.90 (5–23)	0.001 *
Oxygen saturation (%)	96 (25–99)	91 (40–99)	0.001 *

* ALP: Alkaline phosphatase; ALT: Alanine aminotransferase; AST: Aspartate aminotransferase; CK: Creatine kinase; CRP: C Reactive Protein; GGT: Gamma-glutamyl transferase; LDH: Lactate dehydrogenase.

**Table 3:** Corticosteroid and oxygen support therapy.

Therapy		Non-Severe Group	Severe Group	Significance of *p* Value
**Number of Patients *n* (%)**		231 (70.0%)	99 (30.0%)	
Corticosteroid *n* (%)	No	106 (45.9%)	34 (34.3%)	0.052
	Yes	125 (54.1%)	65 (65.7%)	
Oxygen support *n* (%)	No	159 (68.8%)	18 (18.2%)	0.001 *
	Yes	72 (31.2%)	81 (81.8%)	

* Statistically significant value.

**Table 4:** Multinomial regression analysis with severity of disease as dependent variable.

Independent Variables	B	P	OR	95% Confidence Interval	
				Lower Bound	Upper Bound
**Hypertension**	**0.394**	**0.413**	1.483	0.577	3.807
Radiographic finding of pneumonia	0.100	0.880	1.105	0.300	4.068
Localization of pneumonia	1.190	0.094	3.286	0.818	13.203
Neutrophils	−0.843	0.655	0.430	0.011	17.445
Lymphocytes	0.440	0.680	1.552	0.192	12.549
CRP	0.004	0.608	1.004	0.990	1.017
AST	0.005	0.440	1.005	0.992	1.018
ALP	−0.012	0.141	0.988	0.973	1.004
LDH	−0.001	0.308	0.999	0.997	1.101
**CK**	**0.002**	0.032 *	**1.002**	**1.000**	**1.004**
Total proteins	−0.052	0.171	0.949	0.881	1.023
Albumin	−0.029	0.443	0.972	0.903	1.046
Glucose	0.085	0.220	1.089	0.950	1.247
Oxygen saturation	−0.028	0.247	0.973	0.928	1.020
**Oxygen support**	**2.178**	<0.001 *	**8.829**	**3.000**	**25.983**

Significant predictor are show in bold; * denotes statistical significance level at <0.05; OR: odds ratio.

## Data Availability

The data presented in this study are available on request from the corresponding author.
